# Coercive Measures in Psychiatry: A Review of Ethical Arguments

**DOI:** 10.3389/fpsyt.2021.790886

**Published:** 2021-12-14

**Authors:** Marie Chieze, Christine Clavien, Stefan Kaiser, Samia Hurst

**Affiliations:** ^1^Adult Psychiatry Service, Department of Psychiatry, University Hospitals of Geneva, Geneva, Switzerland; ^2^iEH2-Institute of Ethics History Humanities, University of Geneva, Geneva, Switzerland

**Keywords:** coercion, ethics, psychiatry, legitimacy, seclusion, restraint, involuntary hospitalization

## Abstract

**Introduction:** Coercion is frequent in clinical practice, particularly in psychiatry. Since it overrides some fundamental rights of patients (notably their liberty of movement and decision-making), adequate use of coercion requires legal and ethical justifications. In this article, we map out the ethical elements used in the literature to justify or reject the use of coercive measures limiting freedom of movement (seclusion, restraint, involuntary hospitalization) and highlight some important issues.

**Methods:** We conducted a narrative review of the literature by searching the PubMed, Embase, PsycINFO, Google Scholar and Cairn.info databases with the keywords “coercive/compulsory measures/care/treatment, coercion, seclusion, restraint, mental health, psychiatry, involuntary/compulsory hospitalization/admission, ethics, legitimacy.” We collected all ethically relevant elements used in the author's justifications for or against coercive measures limiting freedom of movement (e.g., values, rights, practical considerations, relevant feelings, expected attitudes, risks of side effects), and coded, and ordered them into categories.

**Results:** Some reasons provided in the literature are presented as justifying an absolute prohibition on coercion; they rely on the view that some fundamental rights, such as autonomy, are non-negotiable. Most ethically relevant elements, however, can be used in a balanced weighting of reasons to favor or reject coercive measures in certain circumstances. Professionals mostly agree that coercion is only legitimate in exceptional circumstances, when the infringement of some values (e.g., freedom of movement, short-term autonomy) is the only means to fulfill other, more important values and goals (e.g., patient's safety, the long-term rebuilding of patient's identity and autonomy). The results of evaluations vary according to which moral elements are prioritized over others. Moreover, we found numerous considerations (e.g., conditions, procedural values) for how to ensure that clinicians apply fair decision-making procedures related to coercion. Based on this analysis, we highlight vital topics that need further development.

**Conclusion:** Before using coercive measures limiting freedom of movement, clinicians should consider and weigh all ethically pertinent elements in the situation and actively search for alternatives that are more respectful of patient's well-being and rights. Coercive measures decided upon after a transparent, carefully balanced evaluation process are more likely to be adequate, understood, and accepted by patients and caregivers.

## Introduction

Coercive measures, defined as any measure applied “against the patient's will or in spite of his or her opposition” (1), are commonly used in clinical medical practice, particularly—but not exclusively—in psychiatry (2, 3). The definition of “coercive measures” is complex. These include formal coercion, such as actions limiting freedom of movement (restraint, seclusion), involuntary hospitalization, and forced treatment (4). Informal coercion is another part of the concept and includes any form of influence, pressure, or manipulation of the patient's decisions (5). Formal and informal coercion are conceived as a continuum ranging from persuasion, interpersonal leverage, inducements, and threats to compulsory treatment (5–7). However, this distinction between formal and informal coercion may not be clear, and in practice, there are “gray areas” with sometimes unclear boundaries between a strong incentive and coercion (5, 6). Coercion is also defined according to the subjective perceptions of patients, caregivers, or other stakeholders (lawyers, relatives), which may differ from the objective measures used (8, 9). Different understandings of coercion have an impact on the care provided and are hence important to consider (10, 11). For instance, Trachsel et al. (12, 13) distinguished between curative and palliative psychiatric care based on the definition of palliative care given by the World Health Organization (WHO). From this perspective, the use of coercion may be legitimate or not, depending on whether it is used for a palliative or curative reason (7, 12). In addition, coercive measures with more serious consequences call for greater justifications (6, 12).

Coercive measures infringe on several fundamental rights based on ethical principles (autonomy, freedom of movement and will, bodily integrity). Fundamental rights are guaranteed by the Declaration of Human Rights, the European Convention on Human Rights and Bioethics (14, 15), the Convention on the Rights of Persons with Disabilities (CRPD) (16), and the laws of most countries (1, 17–19); they underpin professional guidelines such as the World Psychiatric Association's Madrid Declaration (20). Despite this, there are crucial variations across (and sometimes within) countries in local legal frameworks and in the ways in which coercion is applied (10, 11, 21).

Allowing coercion involves giving priority to some reasons and principles at the expanse of the fundamental rights and principles infringed by coercion (11, 22). This raises ethical questions about the primacy of the ethical principles used to legitimize coercive measures (4, 23) and about the way in which the principles ought to be properly “balanced” (12, 24, 25). This task is not easy as there is no consensus on the appropriate moral theory to apply (26, 27). For instance, the more we value the personhood of a disturbing patient, the greater the justification required to restrict his/her fundamental rights for the sake of protecting others (care providers, other patients, citizens) (6). Moral theories diverge in the comparative importance they place on different values (e.g., autonomy vs. liberty, community vs. individual rights vs. duties toward others or vs. community rights) (6, 26, 27).

Compared to the 18^th^ century and to many contemporary states, modern Western societies tend to stress the importance of personhood, the individual's place in the community, and the development and prioritization of the principles of self-determination and autonomy (26, 28–30). This societal evolution has changed society's way of thinking about psychiatric care and the identity and vision of psychiatry as a discipline (26). Recent emphasis on the right to autonomy involves questioning the legitimacy of the paternalistic attitude that used to be the norm in medical care. Patient's best interests are increasingly taken as critical elements for deciding upon or justifying coercive measures (26, 28). Consequently, caregivers who assume that they know better than patients what is good for them tend to be considered authoritative and paternalistic. The risk of abuse of power associated with paternalism is now taken seriously (31, 32). The issue of paternalism is hotly debated in medical ethics (28, 33), especially in psychiatry (27, 34) and the context of coercive interventions (4, 6, 7, 35, 36). Distinctions between strong/hard vs. weak/soft paternalism and social (for the good of the community) vs. medical (for the good of the individual) paternalism have been made (4, 7, 27, 28, 33, 34). Moreover, new approaches to ethics of care have emerged, such as relational ethics (37–39), which have helped people to reconsider the individual in a relational context within society. New concepts have appeared such as relational autonomy, which may help us to view coercion in a new light as a practice of care. Indeed, one may try to protect the patient by providing coercive treatment with the aim of facilitating the long-term recovery of autonomy, even if it implies a temporary override of the patient's self-determination (4, 26, 28, 37). This approach of coercion can be conceived of as soft paternalism (7, 40, 41).

Apart from the debates around medical paternalism, ethical discussions regarding the tensions between coercion and the respect of fundamental rights are understudied in psychiatry (4), which is paradoxical since limitations on one's freedom occur more often in medical (including psychiatric) contexts than in other civil or social areas (4, 9, 42). This infringement of freedom, present in coercive care, hinders patients from exercising their autonomy. The notion of autonomy is also complex and diversely understood in the literature, which may affect the evaluation of coercive measures. Studies often associate autonomy with decision-making capacity (4). However, this is not always the case. Autonomy can also be understood as being able to make choices (even people with decision-making capacity might not be autonomous) and to realize one's priorities and values (23, 43, 44). It seems, therefore, that autonomy and decision-making capacity are interdependent but in a complex modality, depending on the individual's temporality and identity (45). This complex relationship between autonomy and decision-making capacity deserves an in-depth analysis (27, 34).

In practice, evaluating the acceptability of a coercive measure often implies evaluating the patients' decision-making capacity (DMC). DMC is an important element to take into account because it refers to the principle of autonomy, and coercions imply overriding patient's autonomy (37, 46–48). In some countries, the laws regulating coercion do not specifically refer to DMC, but in most countries and legal systems, one of the conditions for justifying coercion with a patient is his/her lack of DMC (26, 27, 34, 47). From a legal point of view, DMC involves patient's cognitive abilities (46) and is presumed to exist in adults (49–52). In case of doubt, its evaluation depends on the situation (intervention, time) and on the particular decision that the patient is expected to make (49, 53). However, in detail, the way DMC is regulated differs across countries. In the US, the definition developed by Grisso and Appelbaum is widely used (54): DMC requires understanding, appreciation, reasoning, and making a choice (54–57). In Switzerland, DMC requires understanding (grasping the fundamental elements of the information relevant for a decision), evaluating information (assigning personal meaning to a situation in light of the options available), making a choice (deciding on the basis of the information available and one's own experience, motives and values), and expressing a decision (communicating and defending a choice) (58). In England and Wales, DMC is regulated by the Mental Capacity Act (MCA) of 2005 and requires understanding, retaining, using or weighing information, as well as giving an option (49–51, 59–62). In this model, appreciating and reasoning (Grisso and Appelbaum) are replaced by using or weighing (51, 63–66), but it is not obvious that these terms are equivalent (51). Independent of the particular definition of DMC, a common controversy surrounds its evaluation for people suffering from mental disorders, whether they are decompensated or not (14–16, 31, 67).

Furthermore, the principles of “evidence-based medicine” require scientific evidence of the effectiveness of coercion. When a coercive measure is supposed to guarantee safety (to protect others from a patient's aggressive or disturbing behavior), evidence for risk reduction and forensic or social outcomes should be provided (68). Similarly, when a coercive measure is advocated as a way to treat a patient, it should be demonstrated that coercive measures benefit patients by contributing to their therapy or diagnosis in the short, medium, or long term (69). Indeed, some coercive measures may facilitate the treatment of symptoms or allow for the precise and formal detection of a pathology. The implementation of coercion's efficacy in terms of diagnosis or therapy is much debated, and current scientific data are the subject of controversy (4, 21, 32, 70). From an ethical standpoint, the absence of scientific evidence of therapeutic effectiveness does not necessarily indicate that a type of therapy is illegitimate. This has been discussed in the literature (71) and in psychiatry (69), but little has been written about the particular case of coercion. It therefore seems worthwhile to more closely examine the ethical foundations of legitimate coercive measures.

Being able to justify a coercive measure is not only ethically significant; it also helps to alleviate tensions in clinical practice. Notably, it is critical to distinguish therapeutic and diagnostic goals from safety-based or protective goals (for the patient or others) and to recognize that the latter are not necessarily sufficient reasons to justify coercion. Conversely, safety and risk reduction may be conceived of as a means to achieve therapeutic goals because living in a safe environment favors individual reconstruction. This underlines the importance of thinking about coercion in the global context of long-term care. It would be interesting to see if professionals make these distinctions when discussing the adequacy of coercive measures.

In this article, we map out the ethical reasons used in the literature to assess coercive actions that impose limits to freedom of movement; that is, involuntary admission, seclusion, and restraint. To avoid covering an area that is too broad, we will exclude discussions related to informal coercion and coerced medication. We present the results of our literature search and of our qualitative analysis of the selected articles: We map out the ethical elements used in the literature to justify or reject the use of coercion. We then examine and weigh these elements, and address their relevance for assessing the acceptability of coercion in particular clinical settings. Finally, we highlight a series of important issues.

## Methods

### Literature Search Strategy

In line with a previous systematic review on the efficiency of coercive measures (70), we focused our literature search on formal coercive measures limiting freedom of movement; that is, involuntary admission, seclusion, and restraint. From this perspective, we conducted a narrative (exploratory and non-systematic) review of the literature by searching the PubMed, Embase, PsycINFO, Google Scholar and Cairn.info databases with the keywords: [(coercion) OR (compulsory) OR (seclusion) OR (restraint) OR (coercive measure) OR (involuntary admission) OR (involuntary commitment) OR (coercive care) OR (coercive treatment) OR (compulsory care) OR (compulsory treatment) OR (compulsory admission) OR (compulsory commitment)] AND [(psychiatry) OR (mental health)] AND [(ethics) OR (legitimacy)]. In addition, we cross-checked the references of the selected articles to identify more relevant articles.

### Article Selection

We selected articles by checking the relevance of their titles and abstracts, and then by assessing the remaining full articles to identify those that specifically addressed ethical issues related to formal coercive measures limiting freedom of movement used in a general adult psychiatric inpatient setting. We also included articles on the difficulties inherent in deciding whether to apply coercive measures and the circumstances that may justify their use. We excluded articles on coerced medication, case reports, specifically legal analyses, coercion reduction strategies, and clinical settings outside general psychiatry (addictions, eating disorders, neurological pathologies such as dementia or mental retardation, outpatient commitment, and assisted dying). We also excluded populations with specific outcomes and ethical issues, as they would require their own analyses (i.e., children and adolescents, older patients, forensic institutions, and other medical specialties). We excluded articles that did not describe ethical issues or reasons for or against coercion; for example, those that only mentioned descriptive elements or subjective perceptions on the use of coercion without giving adequate reasons. The first author made the initial selection. In case of doubt or uncertainty, the first and last authors talked about which decision to make.

### Data Extraction and Synthesis

We read the selected articles in detail, took note of all arguments and ethically relevant assertions, and initially classified them into two simple categories: elements in favor of coercion (pro) and elements against coercion (contra). We then decomposed these arguments and assertions into underlying ethical “elements” (values, rights, practical considerations, relevant beliefs about mental disorders, pertinent feelings, expected attitudes, risks of side effects, etc.), using key words and concepts employed by the various authors. We then grouped together elements that were thematically close into different group levels (or families). In regular meetings involving all authors, we reviewed the set of elements to ensure that concepts were clearly defined, that the elements were being used consistently, and that coherence was maintained between higher- and lower-level families of elements. When necessary, we relabeled, merged, or reallocated elements in different families. We successively narrowed our grouping until all elements were classified in a simple, interpretable tree (Table 1).

**Table 1:** Ethical elements used in the literature to discuss the acceptability of coercion, classified under different levels of families (thematic groups).

**Group level 1: Broad families of ethical elements**	**Group level 2: Families of ethical elements**	**Group level 3: Ethical elements**
**Non-negotiable rejection**		
Non-negotiable respect for fundamental rights	Infringement of fundamental, non-negotiable rights	Coercion is an infringement of fundamental rights (freedom of movement and will, autonomy, bodily integrity), to be respected above all (1, 4, 9, 17, 18, 32, 42, 72–80)
	Respect for human dignity	Dignity to be respected, not compatible with coercion (29, 30, 72, 81)
Respect for moral values	Respect for autonomy	Autonomy as an intrinsic human value, to be respected at all costs (4, 17, 29–31, 72, 73, 76, 82, 83)
	Respect for bodily integrity	Violating integrity is prohibited if decision-making capacity is present (1, 17, 73)
	Coercion prohibited if decision-making capacity is present (1, 4, 17, 31, 74)	
Ontology of (a) mental disorder(s)	Mental disorder does not exist; hence, there is no legitimacy for the use of coercion (76, 82, 84, 85)	
**Acceptability under conditions**		
**Elements in favor of coercion**		
Prioritized moral values	Autonomy	Absence of contradiction between autonomy and coercion (86)
		Autonomy to be considered when coercing (1, 9, 86, 87)
		Respect for autonomy not absolute (32)
		Supported autonomy (4)
		Coercion of autonomous patients (1, 17, 77)
	Integrity	Avoid damage to integrity if coercion is used (87, 88)
		Coercion is possible, but integrity may not be violated if autonomy is present (1, 17)
	Respect for dignity	Dignity to be respected, even if patient is coerced (1, 9, 42, 73, 74, 87, 88)
		Coercion can bring a greater perception of dignity (4, 29, 81, 87, 89)
		Dignity as an outcome of coercion assessments (32, 90)
	Benevolence	Institutions and their measures are beneficial (1, 9, 41, 42, 77, 87, 88)
		Coercion is necessary if there is no other possibility of respecting benevolence (1, 4, 9, 17, 18, 31, 41, 42, 47, 68, 77, 81, 88)
		Coercion is needed to protect the patient's interests (paternalism) (4, 9, 18, 29, 31, 32, 36, 40, 42, 47, 48, 73, 75, 77, 80, 86, 87, 89, 91)
		The relational nature of the person allows one to intervene in his/her life (4, 39, 82)
	Non-maleficence	Coercion is needed if there is no other possibility to respect non-maleficence (1, 4, 17, 31, 42, 77, 81)
		Coercion as an alternative to the occurrence of other damage or harm (1, 4, 9, 18, 29, 47, 73–75, 81, 88, 89, 92, 93)
		Not using coercion would be non-assistance to a person in danger (17, 72, 86, 91, 94, 95)
		Less frequent coercion for non-maleficent purposes (17)
	Justice/fairness	Coercion for society's protection and well-being (9, 31, 72)
		Coercion to regulate with justice and fairness (1, 9, 11, 32, 77, 87)
		Individual rights are to be balanced with the common good (74, 81, 91)
		Coercion: Little reference to justice (4)
	Safety	Safety of others (1, 4, 9, 31, 42, 47, 72–74, 81, 88, 89, 91, 93, 96, 97)
		Patient safety (4, 9, 25, 42, 72, 73, 81, 86–89, 91, 93, 98–102)
	Community well-being	Community primacy (good of the many) (1, 29, 42, 72, 73, 81, 103)
Authorized infringement of rights	Overriding fundamental rights and freedoms	Overstepping is allowed under certain circumstances (1, 9, 17, 29, 30, 77, 80, 95)
		Restrictions on freedom to be adapted to the need for treatment (42, 47, 80, 104)
		Dichotomous approach: Rights are respected or not (29)
	Necessary coercion	Unavoidable coercion (1, 9, 17, 42, 73, 88, 93, 96, 105, 106)
		To consider coercion as unethical is too simplistic (9, 17, 32, 73)
	Coercion as naturally present	Coercion is present in daily life (77, 87)
		Coercion is present in daily clinical practice (87)
		Coercion is common in psychiatry (9, 32, 42, 75, 91)
	Legal basis and official recommendations	Legislative norms regulate coercion (1, 4, 9, 17, 22, 37, 41, 42, 47, 73–75, 107, 108)
		Legal standards take the principle of autonomy into account (9, 32, 86)
		Guidelines are based on scientific evidence and ethical outcomes (32, 109)
	Factors influencing the justification of the infringement of rights	Dichotomous approach: Coercion is ethical (or not) (29)
		The conception of freedom changes the justification (74, 77)
Limits to the authorization of coercion	Fundamental rights to be respected, even under coercion (9, 42)	
	Coercion requires ethical justification (1, 17, 31, 78, 110, 111)	
Relational factors to consider	Physician's responsibility	Moral and legal responsibility toward the patient (9, 29, 42, 91, 95, 112)
		Duty of care (25, 99, 103)
		Necessary moral and professional qualities (4, 26, 32, 74, 87)
		Avoid role-based conflicts (1, 38, 86)
	Patient-caregiver interactions	Patient-caregiver relationship (1, 4, 9, 29, 32, 74, 82, 86–89)
		Communication (1, 4, 9, 74, 87, 88, 112–117)
		Caregiver competence (1, 4, 9, 32, 72, 74, 87)
		Debriefing in anticipation of the future (72, 74, 117)
		Lack of health care personnel (72, 89, 93, 109)
	Place of relatives	Relatives to be involved in decisions (1, 4, 11, 87)
		Coercion when relatives are exhausted or overwhelmed (1, 9, 29, 86, 91)
	Beneficial, subjective perceptions	Of caregivers (4, 9, 29, 72–74, 93)
		Of patients (1, 4, 9, 29, 72, 74, 75, 87, 89, 116)
**Elements against coercion**		
Respect for fundamental rights	Infringement of fundamental rights	Rights are to be respected, but can be overstepped in certain circumstances (31, 77, 81, 118)
	Respect for human dignity	Risk of losing dignity with coercion (72, 87, 89, 90, 93)
	Respect for bodily integrity	Coercion as a violation of integrity (73)
Respect for moral values	Respect for autonomy	Coercion affects autonomy (4, 17, 31, 74, 77)
		Respecting autonomy allows one to decrease coercion (4, 31, 77, 100)
		Autonomy to be respected unless there is danger or decision-making incapacity (1, 9, 18, 25, 31, 73, 81, 82, 103, 104, 112, 119–122)
		A mental disorder does not imply a lack of autonomy (4, 9, 24, 31, 32, 76)
	Benevolence	Coercion is contrary to the patient's interests (4, 31)
		Unjustifiable paternalism (4, 9, 29, 31, 32, 73, 76, 91, 104, 123, 124)
		Benevolence often comes first in psychiatry, but is not enough (9, 29, 31, 76, 82)
	Non-maleficence	Coercion is contrary to the principle of non-maleficence (32, 72, 125)
		Prevention of damage in advance is not justifiable (9, 73)
	Safety	Unjustifiable coercion for safety (72, 126)
		Institutionalization for safety is not justifiable (9, 17)
Other elements weighing against coercion	Punitive coercion is unacceptable	Punitive use of coercion (4, 42, 73, 92)
		Punitive perception of coercion (4, 31, 74, 89, 93, 102)
		Difference between punishment and care (18, 32, 82, 126)
	Abuse of power (4, 31, 72, 81, 87, 92)	
	Global process	Coercion prevents an increase in self-esteem and a sense of identity (31, 72)
		No proven efficacy (4, 17, 18, 31, 72, 75, 76, 82, 100, 126)
	The interests of others are put before the patient's interests	Anticipation/comfort of caregivers (1, 9, 31, 73, 89, 93)
		Safety of others is important (6, 9, 18, 40, 42, 73, 76, 89, 127, 128)
		Relatives involved in coercion-related decisions (1, 89, 93)
	Adverse effects of coercion	Negative and traumatic experiences (1, 4, 31, 72, 74, 89, 93, 96, 103, 129–131)
		Risk or aggravation of somatic disorders (4, 17, 31, 75, 87, 89, 92, 93, 125, 130)
		Personality changes (4, 18)
		Coercion can impair decision-making capacity (4)
	Informal coercion	Informal coercion to be considered as coercion in its own right, therefore to be justified (1, 9, 31)
		Informal coercion leaves the patient in a position of no choice (4, 9, 75)
	Formal coercion	More serious consequences require more justification than informal coercion (4, 6, 77)
	Banalization (1, 38, 87)	
Elements of inadmissibility of the justification of coercion	Decision-making capacity	Decision-making incapacity is not sufficient to justify coercion (1, 17, 31, 77, 123)
	Ontology of (a) mental disorder(s)	A mental disorder does not justify coercion (1, 9, 24)
		Identical mental and somatic disorders (18, 32)
Preventing coercion	Alternatives (125)	Coercion not as the first phase of treatment (72)
		Prevention, de-escalation, communication (4, 17, 18, 109, 117)
		Recovery and advance directives (18, 32)
Relational factors to consider	Need for clearer recommendations to respect the patient	Respect for the patient's rights (25, 32, 42)
		Research to be pursued (11, 18, 32, 86, 105, 132)
		Hospital discharge: The gray area of no choice (9, 32)
	Place of psychiatry and psychiatrists	The only discipline where treatment can be provided against the patient's will, so be careful (42, 72, 76)
		Psychiatry is caught between different norms (social, legal, medical), so there are tensions and pressures to be aware of for an ethical decision (26, 42, 87, 91, 110, 123)
	Patient-caregiver relationship	Coercion alters the therapeutic relationship (4, 17, 31, 72, 120, 130)
		Coercion is used to avoid caregiver involvement (72, 89)
		A strong relationship can reduce coercion (4, 10, 32, 93)
	Caregiver competence	Lack of ability to assess dangerousness (81)
		Problem evaluating decision-making capacity (4, 9)
	Subjective perceptions of caregivers	Negative emotions and guilt (4, 17, 74, 87, 89, 93, 132)
		Ambivalent feelings to be analyzed for a patient-centered decision, not the caregiver's self-interest (32, 74)
		Coercion as the omnipotence of caregivers (1, 72, 73)
		Other people's eyes (4, 73)
	Subjective perceptions of patients	Negative experiences of coercion (4, 31, 72, 74, 75, 89, 93, 117, 120)
		Subjective perceptions may differ from objective measures, to be considered for the care and support of the patient (9, 75)
		Principle of the least restrictive measure not applicable because assessment is subjective (74, 133)
**Decision-making procedures for clinicians**		
Conditions for the fair application of coercion	Principles of proportionality and necessity (1, 17, 42, 47, 74, 81, 88, 93, 112)	
	Principle of subsidiarity	Coercion as a last resort (1, 17, 26, 72, 75, 89, 91, 105, 134)
		Least restrictive method (1, 4, 9, 31, 41, 81, 93, 102, 135)
		Choice of method with the best or least negative consequences (29, 74, 77, 81, 136)
		Non-cumulative (but alternative) measures of coercion (1, 17, 81, 88, 93)
	Necessary (but not sufficient) conditions	Severe mental disorder (1, 4, 9, 17, 18, 29, 32, 47, 74, 75, 77, 81, 86, 88, 91)
		Decision-making incapacity (1, 4, 9, 10, 17, 18, 29–32, 74, 75, 77, 81, 86, 89, 91, 105, 137)
		Danger to oneself or others: the right to protection (1, 4, 9, 10, 17, 18, 29, 42, 73–75, 77, 81, 86–89, 91, 105, 113, 115, 134)
		Need for care: the right to treatment (1, 4, 7, 9, 17, 18, 29, 31, 42, 47, 72, 74, 80, 81, 86–88, 91, 92, 94, 105, 124, 137, 138)
		Emergency (1, 17, 18, 42, 81, 87, 91, 105)
		Agitation/violence (1, 17, 73, 81, 89, 91–93, 98, 105, 113, 114, 134)
	Justification according to the overall process of care in which the measure fits	Efficiency (1, 4, 9, 10, 17, 18, 29, 31, 32, 72–75, 77, 81, 86–89, 92, 93, 98, 105, 139)
		Coercion as a care setting (4, 9, 17, 72, 73, 77, 81, 91–93, 135, 139)
		Recovery of autonomy, relational autonomy (4, 9, 17, 26, 37–39, 74, 77, 81, 87, 106, 114)
		Post-acceptance (29, 77, 81)
		Distinction between punishment and care (1, 18, 74)
	Level of justification required	Degree of the coercion continuum (1, 4, 9, 29, 31, 77, 81, 87, 92)
		Degree of influence (4, 29)
		Informal coercion to avoid formal coercion (4, 9, 31, 74, 77, 87)
		Formal coercion varies (42, 47, 75)
	Proper evaluation criteria	Evaluation according to dichotomous criteria (29)
		Individual assessment (1, 31, 42, 47, 74)
		Decision-making capacity assessment (1, 4, 9, 29, 72, 77, 86, 91)
		Intervention assessment (17, 74, 116, 118)
		Assessment of future danger (4, 9, 29, 72, 73, 77, 91)
		Evaluation of the lifting of the measure (1, 47, 73, 81, 87)
		Assessor competencies (1, 29, 74, 87, 115)
Conflicting standards	According to the moral weight at stake (1, 4, 9, 17, 32, 42, 73, 74, 77, 81, 87, 88, 91, 136, 140)	
	Autonomy–safety (1, 4, 9, 32, 42, 74, 87, 91, 93, 97)	
	Benevolence–autonomy (1, 4, 7, 17, 26, 29, 31, 42, 48, 74, 87–89, 91, 93, 136, 137, 140)	
	Non-maleficence-autonomy (1, 4, 17, 42, 74, 88, 91)	
	Beneficence–non-maleficence (4, 87)	
	Benevolence–safety of others (18, 40, 42, 73, 127, 128)	
	Benevolence–equity (4, 9)	
	Risk of abuse of power (4, 31, 74, 87, 130)	
Choice of measure	Balance of benefits and adverse effects (17, 74, 101)	
	Internal caregiver conflicts (4, 7, 25, 42, 74, 89, 93)	
	Value conflicts for relatives (87, 89, 91, 93)	
Evaluation	Patient refusal does not imply decision-making incapacity (1, 9, 17, 24, 29, 31, 74, 77, 87, 91)	
	Mental disorder does not imply decision-making incapacity (1, 4, 9, 18, 31, 75, 77, 81, 86)	
	Evaluation paradoxes	Choice between moral values (4, 87)
		Patients say what caregivers want to hear (29)

## Results

### Literature Search and Selection

We obtained 1,614 articles by searching databases with our research terms. After checking the titles and abstracts, 245 articles remained on the list, including articles in English, French and German. A cross-check of the references allowed us to identify 37 more relevant articles.

We read the 282 articles; 99 articles in English, French and German fulfilled our selection criteria.

### Data Extraction and Synthesis

From our empirical analysis of the retrieved literature, four main categories (“non-negotiable rejection,” “acceptability under conditions: elements in favor,” “acceptability under conditions: elements against,” and “decision-making procedures for clinicians”) and three levels of ethical elements emerged. Table 1 outlines the ethically relevant elements found in the 99 selected articles. Several groups of elements are conducive to an absolute prohibition on coercion when the authors are not willing to balance them against contradictory elements. Other elements speak a priori against the use of coercion, but most authors agree that it is possible to override them in exceptional circumstances when other major moral values can be fulfilled thanks to coercive measures. Coercion is then justified (or not) depending on the respective weight of the elements that speak against or for it. The different ethical elements are presented as applicable components to be considered and weighed against each other when deciding whether to use coercion in clinical practice.

#### Non-negotiable Rejection

In the literature that we found, a minority of authors argue in favor of an absolute ban on the use of medical coercion (see Table 1, main category 1, non-negotiable rejection). The reasons they put forward are that coercion violates fundamental rights (including dignity and integrity), as well as major bioethical principles (autonomy, beneficence, and non-maleficence), which, in their view, do not support any concession. They are not convinced that infringement of these fundamental rights and principles can be legitimately overridden in a psychiatric context, regardless of the reasons provided (31, 72, 73). In those articles, the authors doubt the justification of using ever medical coercive measures to prevent a potential and/or indirect risk to others, thereby limiting this capacity to law enforcement.

#### Acceptability Under Conditions: Elements in Favor

Most authors of the retrieved literature specify that coercion may be an adequate measure, but only in certain circumstances. They then provide details about reasons to favor and reasons to reject coercion. We grouped together all ethically germane elements mentioned as reasons to tolerate coercion (see Table 1, main category 2, acceptability under conditions: elements in favor).

Regarding elements in favor of coercion, some authors point out that moral values such as the patient's autonomy, dignity, and integrity are not necessarily violated by the use of coercion (86, 87). This is the case, for example, if special and benevolent attention is provided during the period of care under coercion, and if coercive measures are used to safeguard the patient's values and long-term wishes (4). According to the Swiss Academy of Medical Sciences (SAMS), autonomy corresponds to respect for a person's free choice and self-determination, dignity corresponds to respect for the whole person, and integrity is primarily understood as respect for bodily integrity (1). However, the literature is far from uniform in the definitions of these ethical concepts (141).

Other moral values are put forward in the literature to justify the occasional use of coercion in different contexts in which the overriding values change (45): major bioethical principles (23) such as beneficence (promoting well-being), non-maleficence (avoiding harm), and justice/equity (the fair distribution of goods among individuals according to need) may counterbalance the infringement of autonomy (1, 9). The safety of patients or others (caregivers, other patients, relatives, society) is another moral value mentioned by some authors to justify coercion (42, 81). The serious disruption of communal life (particularly in hospital units) is a criterion permitted by the Swiss Civil Code for instituting a coercive measure (1). However, as claimed by the SAMS, legal permission does not mean that it is ethically justified, especially if the restriction on the patient's rights is grounded in the aim to ensure the “comfort” of other patients and/or caregivers (inconveniences due to behavior management, noise caused, disturbances, etc.) (1). Protection from violence is a key reason to use coercive measures limiting freedom of movement in psychiatry (2, 3); several authors state this as a reason in favor of such measures, often as the central reason (1, 4, 6, 9, 17, 18, 29, 31, 40, 42, 47, 72–76, 81, 88, 89, 91–93, 96–98, 105, 113, 114, 127, 128, 134). Psychiatry is caught between expectations of care, safety, and even sometimes a degree of social control (judicial authority) (26, 42, 87, 91, 110, 123). This generates tension and pressure that clinicians should be aware of to make an ethical decision. Using coercive actions to prevent a potential and/or indirect risk to others is, in any case, not the same as punishing a person who has already attacked someone else, a role clearly outside the scope of psychiatry (31, 72, 73).

A second group of ethical elements used to justify coercion involves the need to secure a safe, livable environment. Indeed, some authors believe that a peaceful communal life implies that some forms of coercion are necessary (17, 73) and already present (e.g., according to libertarian principles, one can act freely as long as one does not restrain the freedom of others). This implies that everyone should accept a certain form of self-coercion for the sake of communal life. From this perspective, coercion—in the sense of (self-) limitation of a potential omnipotence—is more ordinary than exceptional (77, 87). As pointed out by these authors, it does not seem possible to live in a community without constraints or to enjoy one's rights in an absolute manner without restrictions. Coercion is thus conceived of as a necessary tool for the proper realization of peaceful life in the community. The authors recognize, however, that coercive measures can encroach upon people's rights, and the kinds of coercive measures referred to in medical contexts (particularly in psychiatric ones) are likely to be more intrusive than those needed for an ordinary communal life. Thus, decisions over the need to use intrusive coercive measures must be made on a case-by-case basis (31, 74). The overriding of fundamental rights is regulated by clear laws and official recommendations (1, 32). Nevertheless, this legal dimension is often insufficient for ethical legitimization. Critical situations encountered in clinical practice—especially when the patient's informed consent and right to self-determination are overridden—require a more differentiated evaluation than what the law provides (42). From a legal perspective, only “formal” coercion is considered a serious matter and is precisely regulated (4, 77). That said, “informal” coercion also occurs in medical contexts; it is described as more insidious, often hidden, and more common than one might think; sometimes, caregivers do not realize that they make use of it (9, 31). The authors point out that informal coercion also violates patient's rights. It is thus important to be aware of it and to justify it ethically (1, 4).

Another element used to legitimize coercion is the therapeutic relationship. Patient-caregiver interactions are valued and, in certain circumstances, may be improved by using coercion (4, 72, 74). For instance, this is the case if the patient, in an advanced care planning phase, has agreed with the caregiver that coercion is the best therapeutic means to overcome a crisis. Arguably, coercion can then be seen as a way to enhance the patient's condition and/or therapeutic relationship (89). Many authors consider the relational dimension to be a vital element to take into account when evaluating whether coercion is appropriate (9).

Indeed, when a coercive measure is introduced (regardless of its nature), the authority of the physician (as an individual) exceeds the patient's right to autonomy and self-determination. This overstepping must be defendable as such. Some authors insist that the way in which physicians exercise their authority over their patients must be carefully examined since patients tend to be unduly influenced by health care authorities (1, 91).

For some authors, in the case of conflicting views, the subjective perception of the patient (rather than of the caregiver) is the most relevant factor for deciding whether coercion is beneficial (4, 9, 29).

#### Acceptability Under Conditions: Elements Against

In contrast to the abovementioned list of elements in favor of coercion, the literature describes many ethically relevant elements against the use of coercion (see Table 1, main category 3, acceptability under conditions: elements against). The most obvious reasons to reject coercive measures lie in the fact that they tend to infringe upon fundamental rights such as freedom, autonomy, dignity, and integrity. In some cases, coercion violates beneficence, non-maleficence, or safety (e.g., coercive measures may cause physical harm). These are strong reasons to actively search for alternatives to coercion that make its use unnecessary.

Furthermore, many authors make it clear that coercion, when used for punitive or comfort purposes (for caregivers or others), is not justifiable, except in rare cases that must be carefully substantiated (4, 17, 92). Some authors point out that it is critical to be aware of the risks of abuse of power and of using coercion for the interest of others before that of the patient (31, 73, 89).

Moreover, the frequent, significant side effects of coercion (e.g., post-traumatic stress disorder (PTSD), an increase in psychotic or anxiety symptoms, deep vein thrombosis, strangulation, death, etc.) are described as reasons to refrain from using it (4, 31). In relation to the abovementioned distinction between informal and formal coercion, the absence of decision-making capacity should not facilitate the use of coercion (1, 4, 9). Indeed, an absence of decision-making capacity is necessary to use coercion but is not sufficient (1, 4, 9, 10, 17, 18, 29–32, 74, 75, 77, 81, 86, 89, 91, 105, 137). Decision-making incapacity alone is not enough to coerce someone; paternalism and power abuse would appear (31, 32). Further, some authors consider informal coercion to be just as ethically illegitimate as formal coercion since it impacts patients; while leaving them only the appearance of choice, it is sometimes used in a concealed manner and may even induce stronger negative feelings than formal coercion (9).

In addition, there is the risk of misconstruing crucial notions such as coercion or mental disorders. Some misconceptions cloud the importance of relevant ethical considerations that should be taken into account when assessing the adequacy of coercion. For instance, caregivers who believe coercion to be a usual or normal procedure, or who perceive decision-making capacity as a precondition when speaking about coercion, are unlikely to fairly evaluate the adequacy of coercion (especially when treating patients suffering from mental disorders) (1, 18, 76).

If there are other, more human rights-friendly alternatives, this will constitute a reason not to use coercion (18, 32, 72). Some authors attribute the use of unjustified coercion in psychiatry to an inadequate assumption of authority-with-the-right-to-impose (42, 91). Such an erroneous view of caregiver's authority over the patient is also described as having negative effects on the patient-caregiver relationship (4, 72) and as denoting a lack of competence on the part of caregivers (9, 81).

Another element against the use of coercion is the negative, subjective perceptions reported by caregivers and their patients. These negative feelings accompanying the use of coercion are described in the literature as detrimental or even deleterious. Coercive practices may create more harm than good, making their use highly controversial (73–75).

#### Decision-Making Procedures for Clinicians

In addition to the ethical elements for or against the use of coercion, authors also cover vital procedural values and provide advice for clinicians who need to make decisions (see Table 1, main category 4, decision-making procedures for clinicians).

The authors elaborate on the fair application of coercion, which requires one to take the time to balance the reasons for and against its use. Such an evaluation needs to be undertaken anew in each situation. The authors point out that to make a fair, unbiased evaluation in clinical situations, it is important to identify and consider all elements pertinent to that situation, to *weigh* them properly, and to balance them out. Such a procedure helps one to make ethically acceptable choices about coercive measures (74) (Table 1) and lowers the risk of deciding alone based on gut feelings.

In this context, principles of proportionality, necessity, and subsidiarity are highlighted (81, 93). The overall process of care or the medium-term objective into which the coercion is inserted is explained as making it possible to reinscribe the measure in a broader context than simply managing the crisis, and thus to give it a place as a means of providing care to the patient (18, 77). Furthermore, the degree of justification required can vary according to the extent of coercion or influence used (which can be placed on a continuum) (4, 29). The perspective of the clinician, including his/her moral values, is also considered a relevant aspect for decision-making (29, 74). In addition, there are pertinent conditions that need to be considered in the evaluation; even if taken into account separately, they are not sufficient to make a choice. These conditions include the presence of a severe mental disorder, an incapacity of discernment, being a danger to oneself or others, and/or a need for care (1, 17, 86). Numerous articles point out the use of coercion as a means to recover autonomy based on relational ethics of care and using concepts such as relational autonomy and interpersonal interactions (4, 9, 17, 26, 37–39, 74, 77, 81, 87, 106, 114).

In concrete clinical scenarios, it is necessary to balance the different moral values at stake while making an assessment. Some authors favor certain elements over others: autonomy over safety, beneficence, or non-maleficence, and beneficence over non-maleficence, the safety of others, or fairness (4, 91, 127). When we ignore fundamental elements in the evaluation, we can expect strong moral disagreements between those involved (patients, caregivers, relatives), as well as risks of abuse of power and errors in weighing the benefits and risks of the measure (74, 89). According to some authors, a misconception of patient refusal or mental disorder as an a priori disability also leads to errors in the assessment (31, 77). On the other hand, the caregiver may be caught up in the choice to be made between different moral values (4, 87), and may be misled by the patient's verbalization of what the caregiver expects (29).

## Discussion

Our analysis revealed relevant ethical elements (e.g., rights, values, practical considerations, the feelings of the actors involved, risks of side effects) in the literature for establishing the adequacy of coercion. Among these elements, some tip the balance against the indication for coercion, while others tip the balance in favor of an exceptional indication for coercion. This means that for each case of coercion, we should weigh the appropriate elements.

Our results imply that the main justifications for or against the use of coercion vary according to the moral elements defended (mostly principles such as autonomy, integrity, dignity, beneficence, non-maleficence, equity, and safety) and the relative weight given to them. In the literature, some authors especially stress certain elements (e.g., autonomy) over others (e.g., safety), while some ignore arguably germane elements in their assessment (e.g., patient's need/right to treatment or to protection in crisis situations).

We found strong agreement on the idea that coercive measures should only be used in exceptional circumstances and that in each unique case, they should be legitimized by a fair balance of reasons given the legislation, official recommendations, and all ethical elements pertinent to the situation.

Some authors defend the view of an absolute prohibition on coercion, but this is due to an exclusive focus on fundamental rights that are described as non-negotiable (e.g., the duty to respect autonomy in all circumstances). Most authors recognize that even fundamental rights such as autonomy and self-determination may be infringed upon in exceptional clinical situations. However, this can only be done when all other alternatives that are more respectful of the patient's rights have failed and when coercion helps to achieve the greater good (e.g., cases of immediate risk to the patient or others). The fact that coercion is always presented as a rare option of last resort (often in urgent situations) and that it needs to be thoroughly justified underlines how much importance authors give to patient's autonomy and self-determination. However, we found substantial variability in the evaluation of available alternatives. In some cases, authors do not mention relevant ethical elements in their evaluation. For instance, they may refer autonomy and safety but not benevolence, non-maleficence, or justice (9), or the absolute respect of fundamental rights but not the problem of managing a potential risk to others (31, 72, 73). Justice is often an underevaluated principle in studies on coercion (4). In other cases, they diverge on which ethical element should be prioritized or on how much weight should be attributed to a given element.

Based on the retrieved literature, we propose examining several recurrent points of reflection: the use of different argumentative strategies in the literature; the different categories of models used to assess the adequacy of coercive measures; the need to clarify certain concepts used in psychiatric practice; an analysis of whether there are circumstances or limits beyond which coercion is not acceptable or proportionate; and the importance of considering the manner and means in which coercion is applied. Finally, we propose discussing the use of coercive measures limiting freedom of movement in the context of relational ethics of care through the concepts of relational autonomy and interpersonal relationships.

First, in the retrieved literature, different argumentation strategies legitimize the use of coercion. Many ethical reasons are formulated according to a modality of exception. Coercive measures are considered fundamentally prohibited, but their use may be tolerated in exceptional circumstances detailed in laws, conventions, and national or cantonal regulations. Scientific evidence for the therapeutic effectiveness of coercive measures is highly polemical (70). Their justification and acceptance for use based on recommendations of good practice are most often rooted in expert consensus (32). Another argumentation modality found in the selected articles is the tendency to justify one's action a posteriori based on observed consequences. For example, by justifying the measure by the patient's later subjective reaction (improvement of symptoms; recognition a posteriori of the need for the measure; positive emotions reported) or that of the caregiver (exculpation by justifying the measure via the absence of alternatives to coercion; certainty that otherwise there would have been a dangerous act for the patient or others; that leaving the patient without care would have been deleterious for the latter in the future) (4, 9, 29, 73–75). A third argumentative strategy found in the literature is that of discrediting a posteriori the choice to use coercion by considering that the caregiver lacked competence and misused his/her authority (9, 81). This is an external judgment without necessarily understanding all the issues at stake in real situations, which would require a more detailed analysis of the issues facing the caregiver, who has to make the decision. These argumentation strategies reflect the way in which the authors weigh the various ethical elements at stake.

We would like to highlight one particularly relevant analysis proposed by Torbjörn Tännsjö. In an effort to group argumentative elements together, he worked out four different theoretical models that can be used to assess the adequacy of coercive measures (18, 81, 86). The identified models differ regarding the ethical elements they prioritize:

(1) According to the “need model,” “people suffering from a mental illness, who need medical treatment for it, and who do not assent to treatment, should be coercively treated for their illness” (18).
(2) Based on the “life rescue model,” “a more restricted model would be that only people who suffer from mental illness, who need medical treatment for it, whose lives are put at risk if they are not treated and who do not assent to treatment, should be coercively treated for their illness” (86).
(3) According to the “incompetency model,” “only people who suffer from mental illness, who need medical treatment for it, who are not capable of making an autonomous decision about their medical needs and who do not assent to treatment, should be coercively treated for their illness” (18, 86).
(4) The “full responsibility model” underlines the importance of providing the same care and rights to all patients (e.g., having responsibilities toward patients with mental disorders as any person would ordinarily act” (18). Tännsjö discusses the respective value of these models and favors the “incompetency model” since it respects patient's autonomy as much as possible (18, 86). More research is needed to compare and assess the ethical relevance of these models.

A third interesting point to note is the need, reported by several authors, to clarify concepts used in psychiatric contexts (4, 9, 82). Indeed, we found great disparities in the definitions in the literature, and sometimes, the definitions proposed are unclear (21). For example, coercion is defined according to a dichotomous modality, either as emanating from an external authority or by the limits that one can impose on oneself internally (29, 77); in contrast, it is sometimes defined as a continuum with different degrees of intensity and constraints on freedom (1, 4, 31, 81). Most commonly, a distinction is made between formal, informal, and subjective coercion (4, 17, 77). Other notions need to be clarified for better psychiatric practice, such as person (4, 81, 82), identity (77), freedom (1, 32, 74, 77), or integrity (9, 75). The relevance of using the concept of “dignity” is a matter of debate (90, 142, 143). Regarding coercion, its definition includes the notion of paternalism, which has a pejorative connotation in the common sense, while being understood in a more nuanced way by some authors; “soft paternalism” is seen as a way to take care of patients with mental disorders, at least when the aim is to restore their autonomy (75). While it is important to clarify concepts, the plethora of existing definitions may lead to confusion and misinterpretation. In the domain of psychiatry, more collective work is needed to unify the terminology and concepts used in relation to coercion.

Assuming that coercion can be justified in some circumstances (e.g., when it is the only way to fulfill a fundamental value or right) (9, 17, 42), a crucial question is to identify whether there are limits above which coercion is not proportionate or circumstances in which coercion is not acceptable. Some authors argue that some forms of violations are prohibited whatever other value they may fulfill. For instance, it is never permitted to violate the physical integrity of a competent person (1), to torture a person, or to impose inhuman or degrading treatment (144, 145). The latter risk is the most common in psychiatry. For instance, if a caregiver actually intended to harm, humiliate, or punish a patient with coercive measures, this could not be justified by referring to therapeutic benefits. Clinical settings, however, are full of gray areas of which it is important to be aware. This reflection highlights the relevance and difficulty of virtue ethics: Caregiver's intentions, personality, and responsibility matter when we evaluate the acceptability of a coercive measure (32).

Further, the manner and means of coercion need to be considered while evaluating its adequacy. Even in the case of a purpose that is considered legitimate (e.g., ensuring the safety of, and protecting, the patient or others), the way in which coercion is employed might make it unacceptable. Legislation and professional recommendations provide clear, useful safeguards. There is less ethical conflict about how coercion should be used compared to when it is indicated to use it. Nevertheless, clear, uncontroversial instructions on how to utilize coercion do not alleviate the urgency of developing alternative strategies designed to reduce the use of coercion in the first place (de-escalation techniques, advance directives, a Joint Crisis Plan, a trialogue) (21, 146–148).

Finally, one of the most common arguments in the literature is that the patient's autonomy is a critical element in evaluating the justification of coercive measures limiting freedom of movement (4, 86, 87). Some authors go further: Based on the concept of relational autonomy and on the theory of the social construction of the individual through interpersonal ties, one might perceive coercion as a constructive component of a more global process of the recovery of autonomy (4, 9, 17, 26, 37–39, 74, 77, 81, 87, 106, 114). Rethinking coercion within the relational ethics of care may open up new perspectives on care. This is a promising avenue for future studies.

### Strengths and Limitations

To our knowledge, this article is the first to review the ethical elements mentioned in the literature to justify (or not) the use of coercive measures.

Our approach is based on a non-systematic—and thus non-exhaustive—review of the literature or of all ethical justifications for coercion. Nevertheless, most elements that we found are referred to in several sources, which indicates that we are close to data saturation. Our review thus seems to consider the main reasons for the use of coercion reported in the literature.

The literature we found includes few articles focusing mainly on the ethical issues raised by the use of coercion. Most articles address ethical problems in their discussion section only. It would be worth devoting more conceptual and ethical analysis to these crucial issues to clarify key notions and develop sound, applicable evaluation tools (4, 9, 82). Following the reviewers' comments, we added keywords to our search during the review process and reiterated all the steps of the article selection in the subsequent literature. The articles included based on the revised search strategy resulted in a greater volume of literature, but did not yield additional arguments for our result's structure.

We did not aim to investigate whether there might be different language uses and ethical trends across professional groups of authors (philosophers, lawyers, ethicists, clinicians) or across times (contemporary vs. older articles). Our findings did not reveal clear patterns, but it might be interesting to investigate the matter more closely.

### Conclusions

This narrative review of the literature shows that most authors of the included publications advocate for limiting the use of coercion to well-defined, exceptional circumstances. However, evaluations of acceptability vary and depend on the moral values prioritized by the authors, on the content of local laws, and on official recommendations. When there are no available alternatives that are more respectful of the patient's rights as a whole, clinicians need to be able to justify their choice of using coercion. To this end, it is crucial to be able to identify all relevant ethical elements needed for the assessment. Prior knowledge of existing arguments, conceptual nuances, and controversies allows creating the appropriate distance and making sound decisions when difficult situations arise. In this way, the patient's well-being and autonomy can be preserved as much as possible. Thinking of coercive measures not only as safety and risk reduction methods, but also as part of a process aiming to rebuild identity and autonomy in the medium term, could result in coercion processes that are more acceptable to patients and caregivers. In addition, it remains vital to develop alternatives and to ensure continuous ethical assessment to evaluate whether the negative consequences of a coercive measure can be justified over time.

## Author Contributions

MC helped to develop the research question and strategies, collect and analyze the data, and to edit the main part of the manuscript. SH supervised the project's progress and helped to develop the research question and strategies. SK and CC helped to analyze the data and to build the discussion. All authors contributed to the writing of the manuscript and agreed approved of the final version.

## Funding

This narrative review is part of a research program investigating seclusion and restraint reduction, with funding from the University Hospital of Geneva.

## Conflict of Interest

The authors declare that the research was conducted in the absence of any commercial or financial relationships that could be construed as a potential conflict of interest.

## Publisher's Note

All claims expressed in this article are solely those of the authors and do not necessarily represent those of their affiliated organizations, or those of the publisher, the editors and the reviewers. Any product that may be evaluated in this article, or claim that may be made by its manufacturer, is not guaranteed or endorsed by the publisher.

## References

[1] Swiss Academy of Medical Sciences (SAMS). Assessment of capacity in medical practice. Swiss Med Wkly. (2018) 149:w20058. 10.4414/smw.2019.20058

[2] Fisher WA. Restraint and seclusion: a review of the literature. Am J Psychiatry. (1994) 151:1584–91. 10.1176/ajp.151.11.1584 7943445

[3] Goulet MH Larue C Dumais A. Evaluation of seclusion and restraint reduction programs in mental health: a systematic review. Aggress Violent Behav. (2017) 34:139–46. 10.1016/j.avb.2017.01.019

[4] Hem MH Gjerberg E Husum TL Pedersen R. Ethical challenges when using coercion in mental healthcare: a systematic literature review. Nurs Ethics. (2018) 25:92–110. 10.1177/0969733016629770 26931767

[5] Hotzy F Jaeger M. Clinical relevance of informal coercion in psychiatric treatment-a systematic review. Front Psychiatry. (2016) 7:197. 10.3389/fpsyt.2016.00197 28018248 PMC5149520

[6] Szmukler G Appelbaum PS. Treatment pressures, leverage, coercion, and compulsion in mental health care. J Ment Health. (2008) 17:233–44. 10.1080/09638230802052203

[7] Stoll J Hodel MA Riese F Irwin SA Hoff P Biller-Andorno N. Compulsory interventions in severe and persistent mental illness: a survey on attitudes among psychiatrists in Switzerland. Front Psychiatry. (2021) 12:537379. 10.3389/fpsyt.2021.537379 34113265 PMC8185174

[8] Jaeger S Pfiffner C Weiser P Langle G Croissant D Schepp W. Long-term effects of involuntary hospitalization on medication adherence, treatment engagement and perception of coercion. Soc Psychiatry Psychiatr Epidemiol. (2013) 48:1787–96. 10.1007/s00127-013-0687-x 23604621

[9] Syse A. Coercion in psychiatry. An analytical overview. In: Morrissey JP Monahan J, editors. Research in Community And Mental Health. New York, NY: JAI Press Inc. (1999). p. 81–99.

[10] Sheehan KA. Compulsory treatment in psychiatry. Curr Opin Psychiatry. (2009) 22:582–6. 10.1097/YCO.0b013e328330cd15 19654545

[11] Kallert TW. Coercion in psychiatry. Curr Opin Psychiatry. (2008) 21:485–9. 10.1097/YCO.0b013e328305e49f 18650692

[12] Trachsel M Wild V Biller-Andorno N Krones T. Compulsory treatment in chronic anorexia nervosa by all means? searching for a middle ground between a curative and a palliative approach. Am J Bioeth. (2015) 15:55–6. 10.1080/15265161.2015.1039730 26147269

[13] Trachsel M Irwin SA Biller-Andorno N Hoff P Riese F. Palliative psychiatry for severe persistent mental illness as a new approach to psychiatry? definition, scope, benefits, and risks. BMC Psychiatry. (2016) 16:260. 10.1186/s12888-016-0970-y 27450328 PMC4957930

[14] Gurbai S. Beyond the Pragmatic Definition*?* the right to non-discrimination of persons with disabilities in the context of coercive interventions. Health Hum Rights. (2020) 22:279–92. 32669807 PMC7348457

[15] Martin W Gurbai S. Surveying the Geneva impasse: coercive care and human rights. Int J Law Psychiatry. (2019) 64:117–28. 10.1016/j.ijlp.2019.03.001 31122621 PMC6544564

[16] UN General Assembly. Convention on the Rights of Persons with Disabilities, A/RES/61/106. Treaty Series, vol. 2515 (2006).

[17] Montaguti E Schurmann J Wetterauer C Picozzi M Reiter-Theil S. Reflecting on the reasons pros and cons coercive measures for patients in psychiatric and somatic care: the role of clinical ethics consultation. a pilot study. Front Psychiatry. (2019) 10:441. 10.3389/fpsyt.2019.00441 31281272 PMC6595495

[18] Tannsjo T. The convention on human rights and biomedicine and the use of coercion in psychiatry. J Med Ethics. (2004) 30:430–4. 10.1136/jme.2002.000703 15467070 PMC1733949

[19] Jarrett M Bowers L Simpson A. Coerced medication in psychiatric inpatient care: literature review. J Adv Nurs. (2008) 64:538–48. 10.1111/j.1365-2648.2008.04832.x 19120567

[20] World Psychiatric Association. 1996. Madrid Declaration on Ethical Standards for Psychiatric Practice. Madrid: World Psychiatric Association.

[21] Chieze M Hurst S Sentissi O. Contrainte en psychiatrie: Etat des lieux des preuves d'efficacité. Swiss Arch Neurol Psychiatry. (2018) 169:104–13. 10.4414/sanp.2018.00573

[22] Gill NS Turner K. How the statutory health attorney provision in Mental Health Act 2016 (Qld) is incompatible with human rights. Australas Psychiatry. (2021) 29:72–4. 10.1177/1039856220968406 33211548

[23] Beauchamp T Childress J. Principles of Biomedical Ethics. 5th ed. New York, NY: Oxford University Press (2001).

[24] Matthews E. Autonomy and the psychiatric patient. J Appl Philos. (2000) 17:59–70. 10.1111/1468-5930.00140 11758594

[25] Helmchen H. [Coercion in psychiatry: practical consequences of ethical aspects]. Nervenarzt. (2021) 92:259–66. 10.1007/s00115-020-00998-7 33000288 PMC7943494

[26] Hoff P. Compulsory interventions are challenging the identity of psychiatry. Front Psychiatry. (2019) 10:783. 10.3389/fpsyt.2019.00783 31780962 PMC6851161

[27] Radoilska L. (editor.). Autonomy and Mental Disorder. Oxford: Oxford University Press (2012). p. 328. 10.1093/med/9780199595426.001.0001

[28] Annoni M. Patient protection and paternalism in psychotherapy. In: Trachsel M Gaab J Biller-Andorno N Tekin Ş Sadler JZ, editors. Oxford Handbook of Ethics in Psychotherapy. Oxford University Press (2020). 10.1093/oxfordhb/9780198817338.013.14

[29] Olsen DP. Influence and coercion: relational and rights-based ethical approaches to forced psychiatric treatment. J Psychiatr Ment Health Nurs. (2003) 10:705–12. 10.1046/j.1365-2850.2003.00659.x 15005484

[30] Wilson J. Is respect for autonomy defensible? J Med Ethics. (2007) 33:353–6. 10.1136/jme.2006.018572 17526687 PMC2598284

[31] O'Brien AJ Golding CG. Coercion in mental healthcare: the principle of least coercive care. J Psychiatr Ment Health Nurs. (2003) 10:167–73. 10.1046/j.1365-2850.2003.00571.x 12662333

[32] Kallert TW Mezzich JE Monahan J. Coercive Treatment in Psychiatry: Clinical, legal and ethical aspects. Chichester: Wiley-Blackwell; John Wiley & Sons (2011). 10.1002/9780470978573

[33] Dworkin G. “Paternalism, The Stanford Encyclopedia of Philosophy (Fall 2020 Edition), Edward N. Zalta” (ed.). Available online at: <https://plato.stanford.edu/archives/fall2020/entries/paternalism/>.

[34] Oulis P. “Personal Autonomy in Mental Disorders: Lubomira Radoilska (ed.): Autonomy and Mental Disorder. Oxford: Oxford University Press (2013). p. 328. 10.1007/s11016-013-9756-8

[35] Chieze M Kaiser S Courvoisier D Hurst S Sentissi O Fredouille J. Prevalence and risk factors for seclusion and restraint in old-age psychiatry inpatient units. BMC Psychiatry. (2021) 21:82. 10.1186/s12888-021-03095-4 33557780 PMC7869451

[36] Callahan JC. Liberty, beneficence, and involuntary confinement. J Med Philos. (1984) 9:261–93. 10.1093/jmp/9.3.261 6491555

[37] Series L. Relationships, autonomy and legal capacity: mental capacity and support paradigms. Int J Law Psychiatry. (2015) 40:80–91. 10.1016/j.ijlp.2015.04.010 25982964

[38] Austin W Bergum V Nuttgens S. Addressing oppression in psychiatric care: a relational ethics perspective. Ethical Hum Psychol Psychiatry. (2004) 6:69–78. 15706695

[39] Verkerk M. A care perspective on coercion and autonomy. Bioethics. (1999) 13:358–68. 10.1111/1467-8519.00163 11657245

[40] Sjostrand M Helgesson G. Coercive treatment and autonomy in psychiatry. Bioethics. (2008) 22:113–20. 10.1111/j.1467-8519.2007.00610.x 18251771

[41] Sales EN Candilis PJ. How does law support compassionate mental health practice? AMA J Ethics. (2021) 23:E335–9. 10.1001/amajethics.2021.335 33950829

[42] Guibet-Lafaye C. L'hospitalisation sous contrainte, une source de conflits normatifs. Revue Française Des Affaires Sociales. (2016) 2:35–55. 10.3917/rfas.162.0035

[43] Schwab M Benaroyo L. [The various senses of autonomy and their relevance to clinical practice]. Rev Med Suisse. (2009) 5:2163–5. 19968029

[44] Miller BL. Autonomy & the refusal of lifesaving treatment. Hastings Cent Rep. (1981) 11:22–8. 10.2307/3561339 7298320

[45] Jaworska A. Respecting the margins of agency: Alzheimer's patients and the capacity to value. Philos Public Aff. (1999) 28:105–38. 10.1111/j.1088-4963.1999.00105.x 11657616

[46] Halpern J. When concretized emotion-belief complexes derail decision-making capacity. Bioethics. (2012) 26:108–16. 10.1111/j.1467-8519.2010.01817.x 20497171

[47] Conrady J Roeder T. The legal point of view: comparing differences of legal regulations related to involuntary admission and hospital stay in twelve European countries. In: Kallert TW Torres-Gonzáles F, editors. Legislation on Coercive Mental Health Care In Europe Legal Documents And Comparative Assessment Of Twelve European Countries. Frankfurt am main: Peter Lang (2006). p. 349–74.

[48] Siu BW Fistein EC Leung HW Chan LS Yan CK Lai AC. Compulsory admission in hong kong: balance between paternalism and patient liberty. East Asian Arch Psychiatry. (2018) 28:122–8. 10.12809/eaap1825 30563948

[49] Ryan C Szmukler G Large M. Kings college hospital trust v C: using and weighing information to assess capacity. Lancet Psychiatry. (2016) 3:917–9. 10.1016/S2215-0366(16)30279-6 27692260

[50] Winburn E Mullen R. Personality disorder and competence to refuse treatment. J Med Ethics. (2008) 34:715–6. 10.1136/jme.2007.023341 18827100

[51] Kim SYH. The place of ability to value in the evaluation of decision-making capacity. In: Moseley D Gala G, editors. Philosophy and Psychiatry: Problems, Intersections and New Perspectives. New York, NY: Routledge (2016).

[52] den Hartogh G. Do we need a threshold conception of competence? Med Health Care Philos. (2016) 19:71–83. 10.1007/s11019-015-9646-5 25971689 PMC4805723

[53] Charland LC. Is Mr spock mentally competent? competence to consent and emotion. Philos Psychiatr Psychol. (1998) 5:67–81. 10.1353/ppp.1998.0002

[54] Grisso T Appelbaum PS. Assessing Competence To Consent To Treatment. New York, NY: Oxford University Press (1998).

[55] Grisso T Appelbaum PS. Appreciating anorexia: decisional capacity and the role of values. Philos Psychiatr Psychol. (2006) 13:293–7. 10.1353/ppp.2007.0030

[56] Saks ER Dunn LB Marshall BJ Nayak GV Golshan S Jeste DV. The California scale of appreciation: a new instrument to measure the appreciation component of capacity to consent to research. Am J Geriatr Psychiatry. (2002) 10:166–74. 10.1097/00019442-200203000-00007 11925277

[57] Hermann H Trachsel M Mitchell C Biller-Andorno N. Medical decision-making capacity: knowledge, attitudes, and assessment practices of physicians in Switzerland. Swiss Med Wkly. (2014) 144:w14039. 10.4414/smw.2014.14039 25317865

[58] Swiss Academy of Medical Sciences (SAMS). Assessment Of Capacity In Medical Practice. (2018). p. 29.

[59] Department for Constitutional Affairs. Mental Capacity Act Code of Practice. London: TSO (2007).

[60] David AS Hotopf M Moran P Owen G Szmukler G Richardson G. Mentally disordered or lacking capacity? lessons for management of serious deliberate self harm. BMJ. (2010) 341:587–9. 10.1136/bmj.c4489 20823014

[61] Tan JOA Stewart A Fitzpatrick R Hope T. Competence to make treatment decisions in anorexia nervosa: thinking processes and values. Philos Psychiatr Psychol. (2006) 13:267–82. 10.1353/ppp.2007.0032 18066393 PMC2121578

[62] Gurbai S Fitton E Martin W. Insight under scrutiny in the court of protection: a case law survey. Front Psychiatry. (2020) 11:560329. 10.3389/fpsyt.2020.560329 33061918 PMC7518213

[63] Szmukler G. “Personality disorder” and capacity to make treatment decisions. J Med Ethics. (2009) 35:647–50. 10.1136/jme.2009.030395 19793948

[64] Ayre K Owen GS Moran P. Mental capacity and borderline personality disorder. BJPsych Bulletin. (2017) 41:33–6. 10.1192/pb.bp.115.052753 28184315 PMC5288091

[65] Hubbeling D. Decision-making capacity should not be decisive in emergencies. Med Health Care Philos. (2014) 17:229–38. 10.1007/s11019-013-9534-9 24370815

[66] Hermann H Trachsel M Elger BS Biller-Andorno N. Emotion and value in the evaluation of medical decision-making capacity: a narrative review of arguments. Front Psychol. (2016) 7:765. 10.3389/fpsyg.2016.00765 27303329 PMC4880567

[67] Curk P Gurbai S Freyenhagen F. Removing compliance: interpersonal and social factors affecting insight assessments. Front Psychiatry. (2020) 11:560039. 10.3389/fpsyt.2020.560039 33192677 PMC7533568

[68] Nussbaum AM. Held against our wills: reimagining involuntary commitment. Health Aff. (2020) 39:898–901. 10.1377/hlthaff.2019.00765 32364875

[69] Gupta M. Ethics and evidence in psychiatric practice. Perspect Biol Med. (2009) 52:276–88. 10.1353/pbm.0.0081 19395825

[70] Chieze M Hurst S Kaiser S Sentissi O. Effects of seclusion and restraint in adult psychiatry: a systematic review. Front Psychiatry. (2019) 10:491. 10.3389/fpsyt.2019.00491 31404294 PMC6673758

[71] Hurst S. What 'empirical turn in bioethics'? Bioethics. (2010) 24:439–44. 10.1111/j.1467-8519.2009.01720.x 19438442

[72] Cano N Boyer L. Évolution des pratiques institutionnelles: questions éthiques autour de l'enfermement et de l'isolement. Inf Psychiatr. (2011) 87:589–93. 10.3917/inpsy.8707.0589

[73] Muir-Cochrane E. An exploration of ethical issues associated with the seclusion of psychiatric patients. Collegian. (1995) 2:14–20. 10.1016/S1322-7696(08)60111-0

[74] Widdershoven G Berghmans R. Coercion and pressure in psychiatry: lessons from Ulysses. J Med Ethics. (2007) 33:560–3. 10.1136/jme.2005.015545 17906050 PMC2652789

[75] Hoyer G Kjellin L Engberg M Kaltiala-Heino R Nilstun T Sigurjonsdottir M. Paternalism and autonomy: a presentation of a Nordic study on the use of coercion in the mental health care system. Int J Law Psychiatry. (2002) 25:93–108. 10.1016/S0160-2527(01)00108-X 12071105

[76] Szasz T. Coercion as Cure: A Critical History of Psychiatry. New York, NY: Transaction Publishers (2007).

[77] Wertheimer A. A philosophical examination of coercion for mental health issues. Behav Sci Law. (1993) 11:239–58. 10.1002/bsl.237011030325855820

[78] Dyer C. Unjustified seclusion of psychiatric patients is breach of human rights. BMJ. (2003) 327:183. 10.1136/bmj.327.7408.183-b 12881249 PMC1126571

[79] Szasz T. The case against compulsory psychiatric interventions. Lancet. (1978) 1:1035–6. 10.1016/S0140-6736(78)90753-5 76947

[80] Chodoff P. The case for involuntary hospitalization of the mentally ill. Am J Psychiatry. (1976) 133:496–501. 10.1176/ajp.133.5.496 1267052

[81] Ewuoso CO. Beneficial Coercion in psychiatric care: insights from African Ethico-cultural system. Dev World Bioeth. (2018) 18:91–7. 10.1111/dewb.12137 27990738

[82] Sadegh-Zadeh K. Handbook of Analytic Philosophy of Medicine. 2nd ed. Dordrecht: Springer (2015). 10.1007/978-94-017-9579-1

[83] Lilla M. Les intellectuels et la tyrannie. Commentaire. (2015) 4:775–86. 10.3917/comm.152.0775

[84] Szasz T. The myth of mental illness. Am Psychol. (1960) 15:113–8. 10.1037/h0046535

[85] Engel GL. The need for a new medical model: a challenge for biomedicine. Science. (1977) 196:129–36. 10.1126/science.847460 847460

[86] Tannsjo T. Coercive Care: Ethics of Choice in Health & Medicine. London: Routledge (1999).

[87] Hem MH Molewijk B Pedersen R. Ethical challenges in connection with the use of coercion: a focus group study of health care personnel in mental health care. BMC Med Ethics. (2014) 15:82. 10.1186/1472-6939-15-82 25475895 PMC4269949

[88] Kjellin L Andersson K Candefjord IL Palmstierna T Wallsten T. Ethical benefits and costs of coercion in short-term inpatient psychiatric care. Psychiatr Serv. (1997) 48:1567–70. 10.1176/ps.48.12.1567 9406265

[89] Gutheil TG. Observations on the theoretical bases for seclusion of the psychiatric inpatient. Am J Psychiatry. (1978) 135:325–8. 10.1176/ajp.135.3.325 626222

[90] Plunkett R Kelly BD. Dignity: the elephant in the room in psychiatric inpatient care? a systematic review and thematic synthesis. Int J Law Psychiatry. (2021) 75:101672. 10.1016/j.ijlp.2021.101672 33513475

[91] Pelto-Piri V Kjellin L Lindvall C Engstrom I. Justifications for coercive care in child and adolescent psychiatry, a content analysis of medical documentation in Sweden. BMC Health Serv Res. (2016) 16:66. 10.1186/s12913-016-1310-0 26893126 PMC4759758

[92] Begin S. [Isolation and restraints: review of the literature and focus on their impact and normative component]. Can J Psychiatry. (1991) 36:752–9. 10.1177/070674379103601014 1790523

[93] Lew D. Mesures Physiques Limitant La Liberté De Mouvement. Genève: Comité d'éthique des Hôpitaux Universitaires de Genève (2014).

[94] Prinsen EJ van Delden JJ. Can we justify eliminating coercive measures in psychiatry? J Med Ethics. (2009) 35:69–73. 10.1136/jme.2007.022780 19103948

[95] Muller S. [The influence of the UN convention on the rights of persons with disabilities on the German jurisdiction and legalisation regarding compulsory measures]. Fortschr Neurol Psychiatr. (2018) 86:485–92. 30125919 10.1055/a-0597-2031

[96] Ye J Xiao A Yu L Wei H Wang C Luo T. Physical restraints: an ethical dilemma in mental health services in China. Int J Nurs Sci. (2018) 5:68–71. 10.1016/j.ijnss.2017.12.001 31406804 PMC6626237

[97] Giordano S. For the protection of others. the value of individual autonomy and the safety of others. Health Care Anal. (2000) 8:309–19. 10.1023/A:1009404321431 11186028

[98] Savage L Salib E. Seclusion in psychiatry. Nurs Stand. (1999) 13:34–7. 10.7748/ns1999.09.13.50.34.c2670 10661207

[99] Bourbon-Vincent M. [Freedom of movement: a basic right, a regulated restriction]. Soins Psychiatr. (2017) 38:17–20. 10.1016/j.spsy.2017.03.004 28476250

[100] Borecky A Thomsen C Dubov A. reweighing the ethical tradeoffs in the involuntary hospitalization of suicidal patients. Am J Bioeth. (2019) 19:71–83. 10.1080/15265161.2019.1654557 31557114

[101] Wagner M. [Isolation and restraint in psychiatry]. Rev Infirm. (2019) 68:28–30. 10.1016/j.revinf.2019.03.010 31147072

[102] Muir-Cochrane EC Holmes CA. Legal and ethical aspects of seclusion: an Australian perspective. J Psychiatr Ment Health Nurs. (2001) 8:501–6. 10.1046/j.1351-0126.2001.00413.x 11842477

[103] Meier-Allmendinger D. [Compulsory hospital admission - coercive measures in medical care]. Ther Umsch. (2009) 66:595–9. 10.1024/0040-5930.66.8.595 19653155

[104] McLachlan AJ Mulder RT. Criteria for involuntary hospitalisation. Aust N Z J Psychiatry. (1999) 33:729–33. 10.1080/j.1440-1614.1999.00636.x 10544998

[105] Purgato M Valimaki M Whittington R Clifton A Coutinho ES Huf G. 'Consent rituals' in evaluation of coercive care. Evid Based Ment Health. (2013) 16:32–3. 10.1136/eb-2012-101190 23426959

[106] Geller JL. Patient-centered, recovery-oriented psychiatric care and treatment are not always voluntary. Psychiatr Serv. (2012) 63:493–5. 10.1176/appi.ps.201100503 22549535

[107] Little JD. Coercive care and human rights; a complex juxtaposition - part 2. Australas Psychiatry. (2019) 27:438–40. 10.1177/1039856219879522 31545089

[108] Little JD. Coercive care and human rights; a complex juxtaposition - part 1. Australas Psychiatry. (2019) 27:435–7. 10.1177/1039856219852283 31107099

[109] Zheng C Li S Chen Y Ye J Xiao A Xia Z. Ethical consideration on use of seclusion in mental health services. Int J Nurs Sci. (2020) 7:116–20. 10.1016/j.ijnss.2019.10.001 32099869 PMC7031114

[110] Wynn R. Coercion in psychiatric care: clinical, legal, and ethical controversies. Int J Psychiatry Clin Pract. (2006) 10:247–51. 10.1080/13651500600650026 24941142

[111] Vaught W Lamdan RM. An ethics committee explores restraint use and practices. HEC Forum. (1998) 10:306–16. 10.1023/A:1008808717281 10338983

[112] Liegeois A Eneman M. Ethics of deliberation, consent and coercion in psychiatry. J Med Ethics. (2008) 34:73–6. 10.1136/jme.2006.019695 18234941

[113] Hopton J. Control and restraint in contemporary psychiatric nursing: some ethical considerations. J Adv Nurs. (1995) 22:110–5. 10.1046/j.1365-2648.1995.22010110.x 7560517

[114] Myers S. Seclusion: a last resort measure. Perspect Psychiatr Care. (1990) 26:24–8. 10.1111/j.1744-6163.1990.tb00313.x 2255580

[115] Berge T Bjontegard KS Ekern P Furan M Landro NI Larsen GJS. Coercive mental health care - dilemmas in the decision-making process. Tidsskr Nor Laegeforen. (2018) 138:12. 10.4045/tidsskr.17.0338 30132604

[116] Lorem GF Hem MH Molewijk B. Good coercion: patients' moral evaluation of coercion in mental health care. Int J Ment Health Nurs. (2015) 24:231–40. 10.1111/inm.12106 25394674

[117] Krieger E Moritz S Weil R Nagel M. Patients' attitudes towards and acceptance of coercion in psychiatry. Psychiatry Res. (2018) 260:478–85. 10.1016/j.psychres.2017.12.029 29287276

[118] Petrini C. Ethical considerations for evaluating the issue of physical restraint in psychiatry. Ann Ist Super Sanita. (2013) 49:281–5. 10.4415/ANN_13_03_08 24071608

[119] Chin HP. Detention, capacity, and treatment in the mentally ill-ethical and legal challenges. Camb Q Healthc Ethics. (2019) 28:752–8. 10.1017/S0963180119000690 31526417

[120] Deutsche Gesellschaft fur Psychiatrie und Psychotherapie PuNeV. [Respect for self-determination and use of coercion in the treatment of mentally ill persons: an ethical position statement of the DGPPN]. Nervenarzt. (2014) 85:1419–31. 10.1007/s00115-014-4202-8 25388831

[121] Griffith R Tengnah C. Deprivation of liberty safeguards. Br J Community Nurs. (2008) 13:532–7. 10.12968/bjcn.2008.13.11.31527 18981971

[122] Monahan J. John Stuart mill on the liberty of the mentally ill: a historical note. Am J Psychiatry. (1977) 134:1428–9. 10.1176/ajp.134.12.1428 335904

[123] Dresser R. Involuntary confinement: legal and psychiatric perspectives. J Med Philos. (1984) 9:295–9. 10.1093/jmp/9.3.295 6491556

[124] Rosenman S. Psychiatrists and compulsion: a map of ethics. Aust N Z J Psychiatry. (1998) 32:785–93. 10.3109/00048679809073867 10084342

[125] Stastny P. Involuntary psychiatric interventions: a breach of the Hippocratic oath? Ethical Hum Sci Serv. (2000) 2:21–41. 15586452

[126] Zaami S Rinaldi R Bersani G Marinelli E. Restraints and seclusion in psychiatry: striking a balance between protection and coercion. critical overview of international regulations and rulings. Riv Psichiatr. (2020) 55:16–23. 10.1708/3301.32714 32051621

[127] Sprumont D Borghi M Guillod O Aebi-Muller R. ASSM clarification juridique. Bulletin Des Médecins Suisses. (2016) 97:845–7. 10.4414/bms.2016.04645

[128] Gravier B Eytan A. [Ethical issues in psychiatry under coercion]. Rev Med Suisse. (2011) 7:1806, 1808-11. 22016935

[129] Steinert T Bergbauer G Schmid P Gebhardt RP. Seclusion and restraint in patients with schizophrenia: clinical and biographical correlates. J Nerv Ment Dis. (2007) 195:492–6. 10.1097/NMD.0b013e3180302af6 17568297

[130] Paradis-Gagne E Pariseau-Legault P Goulet MH Jacob JD Lessard-Deschenes C. Coercion in psychiatric and mental health nursing: a conceptual analysis. Int J Ment Health Nurs. (2021) 30:590–609. 10.1111/inm.12855 33694266

[131] Szasz TS. The danger of coercive psychiatry. Am Bar Assoc J. (1975) 61:1246–8. 11664493

[132] E WH Ruud T Hynnekleiv T. Ethical challenges of seclusion in psychiatric inpatient wards: a qualitative study of the experiences of Norwegian mental health professionals. BMC Health Serv Res. (2019) 19:879. 10.1186/s12913-019-4727-4 31752958 PMC6873436

[133] Gutheil TG Appelbaum PS Wexler DB. The inappropriateness of “least restrictive alternative” analysis for involuntary procedures with the institutionalized mentally ill. J Psychiatry Law. (1983) 11:7–17. 10.1177/009318538301100102 11658420

[134] Tarbuck P. Mental health. ethical standards and human rights. Nurs Stand. (1992) 7:27–30. 1467212

[135] Olsen DP. Toward an ethical standard for coerced mental health treatment: least restrictive or most therapeutic? J Clin Ethics. (1998) 9:235–46. 10029824

[136] Levenson JL. Psychiatric commitment and involuntary hospitalization: an ethical perspective. Psychiatr Q. (1986) 58:106–12. 10.1007/BF01064051 3562678

[137] Vollmann J. [Compulsory treatment in psychiatry: an ethical analysis of the new legal regulations for clinical practice]. Nervenarzt. (2014) 85:614–20. 10.1007/s00115-013-3866-9 23979362

[138] Carroll MA. The right to treatment and involuntary commitment. J Med Philos. (1980) 5:278–91. 10.1093/jmp/5.4.278 7205095

[139] Clare AW. In defence of compulsory psychiatric intervention. Lancet. (1978) 1:1197–8. 10.1016/S0140-6736(78)90980-7 77956

[140] Chodoff P. Involuntary hospitalization of the mentally ill as a moral issue. Am J Psychiatry. (1984) 141:384–9. 10.1176/ajp.141.3.384 6703103

[141] Schroeder M. Value Theory. The Stanford Encyclopedia of Philosophy, (Spring 2021 Edition), Edward N. Zalta (editor.). Available online at: <https://plato.stanford.edu/archives/spr2021/entries/value-theory/>.

[142] Macklin R. Dignity is a useless concept. BMJ. (2003) 327:1419–20. 10.1136/bmj.327.7429.1419 14684633 PMC300789

[143] Pinker S. The Stupidity of Dignity. New Republic. (2008).

[144] Cour Européenne des Droits de l'Homme. Convention de sauvegarde des droits de l'homme et des libertés fondamentales. Rome (1950).

[145] Assemblée Générale des Nations Unies. Convention contre la torture et autres peines ou traitements cruels, inhumains ou dégradants. 0105. New York, NY (1984).

[146] Amering M. Trialog-an exercise in communication between consumers, carers and professional mental health workers beyond role stereotypes. Int J Integr Care. (2010) 10:e014. 10.5334/ijic.484 20228911 PMC2834905

[147] Khazaal Y Chatton A Pasandin N Zullino D Preisig M. Advance directives based on cognitive therapy: a way to overcome coercion related problems. Patient Educ Couns. (2009) 74:35–8. 10.1016/j.pec.2008.08.006 18829211

[148] Hankin CS Bronstone A Koran LM. Agitation in the inpatient psychiatric setting: a review of clinical presentation, burden, and treatment. J Psychiatr Pract. (2011) 17:170–85. 10.1097/01.pra.0000398410.21374.7d 21586995

